# A Rare Case of Adult Onset Intussusception Complicated By Narcotic Dependence

**DOI:** 10.7759/cureus.964

**Published:** 2017-01-08

**Authors:** Saira J Khan, Ashley M Desmarais, Bellal Joseph, Richard Amini

**Affiliations:** 1 College of Medicine, University of Arizona; 2 Department of Surgery, University of Arizona; 3 Emergency Medicine, University of Arizona

**Keywords:** intussusception, narcotic dependence, gastric bypass, delayed presentation

## Abstract

This report describes a rare case of adult intussusception in a patient with a history of a Roux-en-Y gastric bypass procedure; complicated by a history of narcotic abuse, methadone dependence, and methamphetamine abuse. Adult patients who have undergone a Roux-en-Y gastric bypass procedure may be at an increased risk of developing intussusception, and clinicians involved in their care should be aware of this potential complication.

## Introduction

Historically, intussusception has been known primarily as a pediatric diagnosis and uncommon in the adult patient [1]. Diagnosis and definitive care are often delayed due to its nonspecific symptoms including episodic nausea, vomiting, and abdominal pain [2]. Adult onset intussusception may result from Roux-en-Y gastric bypass procedure (RYGBP) and can present as a small bowel obstruction [3].

Nonmedical and medical hydrocodone use has more than doubled from 1999 to 2011. During this same time frame, oxycodone consumption increased by almost 500 percent. As opiate addiction rates continue to increase, the associated morbidity and mortality increase as well [4]. Methadone therapy, heroin abuse, and any other narcotic drug use are well known to adversely affect the motility of the intestines. Opiates are not only highly effective analgesics but also cause intestine dysmotility by affecting the μ-receptors in all three intestinal layers [5]. By decreasing gastrointestinal neuronal activity, reducing peristaltic activity, and delaying the transit of contents through the intestines, narcotic use may increase the risk of intussusception already posed by RYGBP.

## Case presentation

A female patient presented to the emergency department (ED) with a chief complaint of worsening nausea and abdominal pain. The patient was evaluated three days prior in the ED for acute onset abdominal pain but left against medical advice prior to obtaining imaging studies. Past medical, surgical, and social histories were significant for surgical history of RYGBP for weightloss six years prior and an exploratory laparoscopy with drain placement for a marginal gastrojejunal ulcer four months prior. The patient was also receiving methadone treatment for a previous history of narcotic use. The patient had a remote history of intravenous heroin abuse but reported two years sobriety and compliance with methadone for numerous years. In triage her vital signs were: temperature 36°C, blood pressure 132/86 mm Hg, heart rate 107 beats/min, respiratory rate 16 breaths/min, SpO2 100% on room air.

On examination, the patient was noted to be thin and malnourished. She was distressed, screaming in pain, writhing in bed, and difficult to redirect or examine. The patient was responding to external stimulus, using inappropriate words, and was repeatedly trying to get out of bed. The patient was protecting her airway; her pulmonary examination demonstrated tachypnea and equal bilateral breath sounds without wheezing or rhonchi. Her cardiac examination was significant for tachycardia, and the patient's Glasgow Coma Score (GCS) was 13. The abdominal examination was significant for severe tenderness to palpation of the upper quadrants with guarding throughout. There was no rebound tenderness.

The patient was given fentanyl and hydromorphone for pain control, but continued to be agitated. She consistently attempted to get out of bed, was difficult to redirect, and repeat abdominal examinations could not be conducted. At that point, the patient required chemical sedation with lorazepam and haloperidol as she was acutely ill and impeding life-saving interventions. Repeat examination of the patient after chemical sedation demonstrated a somnolent patient with a GCS of 7 and was hypoxic and unable to protect her airway. The patient was immediately intubated for airway protection.

**Figure 1:**
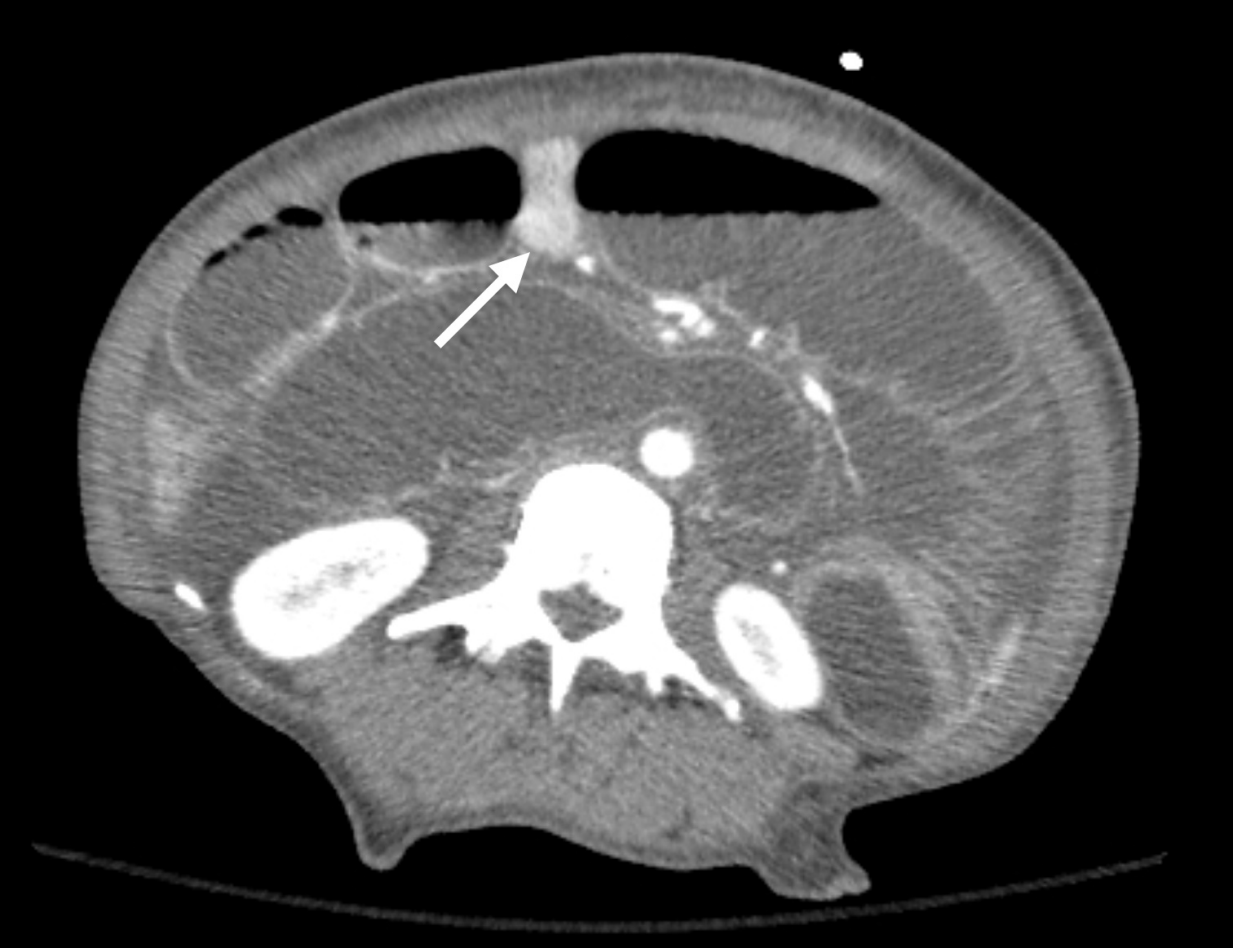
Axial CT Scan CT scan demonstrating dilated loops of the bowel and intussuscepted bowel (white arrow) at the jejunojejunostomy.

Initial laboratory findings demonstrated normal serum leukocyte, lactate, and platelet levels. Urine toxicology screen was positive for opioids, amphetamines, and benzodiazepines. Computed tomography (CT) of the abdomen and pelvis demonstrated small bowel intussusception at the jejunojejunostomy with findings of advanced ischemic changes (Figure 1).

The general surgery team was consulted and immediately took the patient to the operating room. Exploratory laparotomy revealed intussuscepted bowel with distention of the afferent limb and findings of advanced ischemic changes.

The Surgeons performed a small bowel resection of the necrosed bowel and small bowel anastomosis. She was discharged home ambulatory, afebrile, and tolerating a normal diet on postoperative day six. No reference to the patient's identity was made at any stage during data analysis or in this report.

## Discussion

Although uncommon, adult onset intussusception should be included in the differential diagnosis in patients with a history of RYGBP presenting with symptoms concerning for small bowel obstruction [2]. Intussusception can lead to serious complications including ischemia and necrosis of the affected segment, as was seen in this case [3]. Intussusception occurring after laparoscopic RYGBP is considered a rare complication. The most common portion of RYGBP involved in the adult intussusception is the jejunojejunostomy area [6]. Motility studies have shown that RYGBP generally has a higher incidence of dysmotility disorders, called Roux stasis syndrome, and seen in about 30% of patients [7]. Furthermore, small bowel obstruction is reported to occur in 0.2-4.5% of patients who undergo RYGBP and can occur months to years after the procedure [3].

Little is known about the mechanism causing post-RYGBP intussusception; however, a literature review suggests that underlying causes include jejunal spasm with irregular motility, a long afferent loop, an efferent loop with increased motility, widening of the upper jejunum, or increased intra-abdominal pressure [8]. Suggested mechanical causes include adhesions to the mesocolon and jejunal stenosis with obstruction causing antiperistalsis and other abnormal motilities [9]. Despite the mechanism, the dynamics are easily illustrated: as peristalsis in the afferent loop--or antiperistalsis in the efferent loop--continues, the jejunum continues to be pushed into the stoma [8].

In this case, the patient's nausea and vomiting were likely caused by the need to decompress the Roux limb due to the small bowel obstruction at the jejunojejunostomy level, further complicated by the patient's narcotic use and drug abuse. While opiate use has been linked to small bowel obstruction due to decreased intestinal motility, literature on the role of opiate abuse in developing intussusception is sparse. The normal peristaltic function of the small intestine is to transport chyme at a rate of 1-2 cm/second [6]. Through different mechanisms, both RYGBP and opiate use can contribute to decreased gastric mobility [10]. As a result, since gastric dysmotility is implicated in the development of intussusception post RYGBP, it is reasonable to conclude that agents--such as opiates--can act synergistically and increase the risk of intussusception.

## Conclusions

Adult patients who have undergone a Roux-en-Y gastric bypass procedure may be at an increased risk of developing intussusception, and clinicians involved in their care should be aware that agents, such as opiates, can act synergistically to increase the risk of intussusception.
